# Association of uncoupling protein (*Ucp*) gene polymorphisms with cardiometabolic diseases

**DOI:** 10.1186/s10020-020-00180-4

**Published:** 2020-05-25

**Authors:** Anna E. Pravednikova, Sergey Y. Shevchenko, Victor V. Kerchev, Manana R. Skhirtladze, Svetlana N. Larina, Zaur M. Kachaev, Alexander D. Egorov, Yulii V. Shidlovskii

**Affiliations:** 1 Laboratory of Gene Expression Regulation in Development, Institute of Gene Biology, Russian Academy of Sciences, Moscow, Russia; 2 I.M. Sechenov First Moscow State Medical University, Ministry of Health of the Russian Federation, Moscow, Russia

## Abstract

The hereditary aspect of obesity is a major focus of modern medical genetics. The genetic background is known to determine a higher-than-average prevalence of obesity in certain regions, like Oceania. There is evidence that dysfunction of brown adipose tissue (BAT) may be a risk factor for obesity and type 2 diabetes (T2D). A significant number of studies in the field focus on the UCP family. The *Ucp* genes code for electron transport carriers. UCP1 (thermogenin) is the most abundant protein of the UCP superfamily and is expressed in BAT, contributing to its capability of generating heat. Single nucleotide polymorphisms (SNPs) of *Ucp1–Ucp3* were recently associated with risk of cardiometabolic diseases. This review covers the main *Ucp* SNPs A–3826G, A–1766G, A–112C, Met229Leu, Ala64Thr (*Ucp1*), Ala55Val, G–866A (*Ucp2*), and C–55 T (*Ucp3*), which may be associated with the development of obesity, disturbance in lipid metabolism, T2D, and cardiovascular diseases.

## Background

In recent years, there has been a growing interest in the genetics of obesity as one of the major risk factors for cardiovascular diseases and type 2 diabetes (T2D). The prevalence of obesity is increasing worldwide, and being overweight often becomes a chronic, relapsing disease.

Genetic predisposition to obesity can play important role in its etiology together with environmental influences. This idea was confirmed in studies involving twins (Herskind et al. 1996) and adopted children (Stunkard et al. 1986). A high heritability of body mass index (BMI) was shown in 1233 Danish adults, females were found to have a greater heritability than males, and the BMI heritability in males appeared to increase with age (Herskind et al. 1996). A large study including 540 adoptees and their biological and adoptive parents found a significant relationship between the weight class (thin, median weight, overweight, and obese) of the adoptees and the BMI of their biologic parents, but no relationship was observed with the weight class of the adoptive parents (Stunkard et al. 1986)*.* According to published studies, heritability explains most of the variation (61–80%) in BMI, a primary measure of adiposity (Nan et al. 2012), reflecting a high genetic contribution to the etiology of overweight and obesity. This seems promising for those who begin to study the genetic aspect of obesity. Based on the special role that brown adipose tissue (BAT) plays in systemic metabolism, the *Ucp* genes could be of importance. Five homologous genes of the *Ucp* family have been described in humans.

All of the UCP proteins are located on the inner membrane of mitochondria and act to reduce the proton gradient to a greater or lesser degree and thereby to decrease the mitochondrial electrochemical potential (Cline et al. 2001; Gong et al. 1997; Krauss et al. 2002; Mao et al. 1999; Paulik et al. 1998; Sanchis et al. 1998). Their expression is tissue specific: UCP4 and UCP5 play their role in cells of the central nervous system (Ramsden et al. 2012); UCP3 is present in skeletal muscle cells (Boss et al. 1997), BAT (Vidal-Puig et al. 1997), and cardiomyocytes (Razeghi et al. 2002); and UCP2 is found in considerable amounts in white adipose tissue, skeletal muscle, the heart, the lung, the spleen, the thymus, cells of the immune system, and vascular cells and is expressed in lower amounts in the brain, liver, and kidney (Fisler and Warden 2006; Pierelli et al. 2017). UCP1, or thermogenin, is specific to BAT and determines its main function, which is heat generation. Thermogenesis in BAT is essential for the majority of mammals and especially for true hibernators (marmots, hamsters, and bats), non-hibernating small rodents (rats and mice) (Cannon and Nedergaard 2004) and rabbits (Hardman et al. 1969); certain large mammals such as horses (Ertelt et al. 2014), bears (Nedergaard and Cannon 1990) and cetaceans (Hashimoto et al. 2015); and primates (Génin et al. 2003), including humans (Ricquier 2016). BAT is vital in human newborns because their thermoregulation is not developed enough to allow efficient cold adaptation. BAT activation is a promising approach to controlling obesity and, therefore, diabetes and cardiovascular diseases and has attracted substantial interest of researchers after important milestones were achieved. First, the presence of brown fat cells in a human adult body was proved (Heaton 1972). Second, animal experiments showed that BAT is capable of eliminating excess calories consumed with excess food (Rothwell and Stock 1997). UCP1, being the ultimate marker of BAT, is of interest for the genetics of obesity and the focus of this review.

### Brown adipose tissue and UCP1: general characteristics

Apart from muscles, the liver, the pancreas and the brain, BAT has emerged as an important metabolic organ that is evolutionarily designed to generate heat and thus protect mammals from hypothermia (Cannon and Nedergaard 2004). BAT and its putative role in hibernation were first described in 1551 by the Swiss naturalist Konrad Gessner, who found BAT in the interscapular area of marmots (Cannon and Nedergaard 2008). Different functions were assigned to BAT since that time. In 1670, BAT was incorrectly characterized as part of the thymus. Later, in 1817, BAT was considered to be a ductless gland with hemopoietic properties. Since 1863, BAT was classified as a form of adipose tissue that functions as a nutrient pool. In 1902, the endocrine function of BAT was addressed again (Rasmussen 1923). The thermogenic function of BAT was demonstrated in the 1960s by in vivo studies in rodents, and then brown fat depots were similarly found in human newborns (Ricquier 2016). Brown fat contributes to nonshivering thermogenesis (Lichtenbelt 2012) in the infant’s body and prevents hypothermia until the body is capable of independent thermoregulation. The blood flowing through brown fat depots gets warmed up and then increases the temperature of vital organs: the brain, the heart, the lung, the kidney, and even skeletal muscles (Oppert et al. 1994; Ricquier 2011). Heat is not generated by contractile activity in brown adipocytes, which act mostly by increasing the metabolic rate and oxygen consumption of the body. The content of brown fat decreases significantly as the body ages, but there is evidence that small amounts of BAT still remain in adults (Cannon and Nedergaard 2004). The presence of BAT in adults was confirmed in many studies (Heaton 1972; Huttunen et al. 1981). Although the BAT contribution was thought to decrease with age, it was proved about a decade ago that BAT found in human adults is functionally active (Cypess et al. 2009; van Marken Lichtenbelt et al. 2009; Virtanen et al. 2009). This finding renewed interest to BAT as an important metabolic organ in humans.

BAT differs from white adipose tissue in performing other functions and having a different morphological structure and anatomical localization. Brown adipocytes are smaller, and their cytoplasm contains a large number of small lipid droplets, while white fat cells contain one large lipid droplet (Cannon and Nedergaard 2004). Brown fat cells have many mitochondria, which ensure their high metabolic activity. The innervation and vascularization of BAT are several times higher than in white adipose tissue, and BAT is therefore capable of responding faster to a decrease in ambient temperature and delivering heat to vital organs (Saely et al. 2011). While white adipose tissue is distributed almost throughout the body, BAT is located in the supraclavicular, cervical, axillary, and mediastinal regions (Lee et al. 2013; Saely et al. 2011; Sampath et al. 2016).

Today, fluorine-18-fluorodeoxyglucose (18F-FDG) positron emission tomography (PET) is the main tool for visualizing BAT in humans (Sampath et al. 2016). BAT, which actively generates heat, accumulates the tracer more intensely than surrounding tissues as a result of a higher glucose uptake. PET studies were conducted in various populations, and metabolically active BAT was found in 1.2–7.2% of adult subjects (Au-Yong et al. 2009; Zhang et al. 2014b). However, the studies were performed in different climatic conditions and the subjects differed in age and BMI, which is a ratio of body weight to height squared (kg/m^2^). People with active BAT were younger and had lower BMI values (Yoneshiro et al. 2011; Zhang et al. 2014b). In addition, the probability to detect BAT activation is higher in subjects with previous exposure to low air temperatures (Sampath et al. 2016). It is interesting to note that active BAT is more frequent in women than in men (Cypess et al. 2009). Thus, functionally active BAT may contribute to maintaining the normal body weight in some individuals, and exposure to cold triggers a physiological mechanism that induces nonshivering thermogenesis (van Marken Lichtenbelt et al. 2009).

The therapeutic potential of BAT is promising. Despite its low content in the body, BAT can exert a significant metabolic effect. In adults, BAT contributes 1–5% to overall energy metabolism of the body and defects in its function might lead to an excess weight gain of 1–2 kg per year (Urhammer et al. 1997). Scientific interest in BAT is reasonable due to the possibility to develope treatment for metabolic disorders. In fact, it was noted that, white adipose tissue can transdifferentiate into BAT during chronic cold exposure and that BAT loses its capability of thermogenesis and transdifferentiates into white adipose tissue during a prolonged positive energy balance (Barbatelli et al. 2010; Cinti et al. 1997; Rosenwald et al. 2013). Constant sympathetic stimulation was shown to result in the formation of thermogenin-expressing loci in white adipose tissue (Cinti et al. 1997). This process is called browning (Bartelt and Heeren 2014). Beige adipocytes, which are also known as brown-like-in-white cells, could be found within white adipose tissue. Beige adipocytes possess multiple lipid droplets and a high number of mitochondria, thus morphologically resembling brown adipocytes (Phillips 2019). Since beige cells express *Ucp1* similarly to BAT cells, they are capable of thermogenesis (Harms and Seale 2013; Rui 2017). Brown-like loci were first identified in adult mice and were found to emerge in white adipose tissue of the parametrium in response to cold acclimation (Young et al. 1984). Beige adipocytes predominantly originate from the Myf5 (myogenic factor 5)-negative lineage, like white adipocytes, while brown adipocytes arise from Myf + precursors (Phillips 2019). Recent studies in the field revealed that the exercise-induced myokine irisin can trigger the formation of beige adipocytes within white fat pads (Seale et al. 2007). A beige adipocyte-specific transcriptional pattern (*Ucp1*, *Ppargc1α*, *PRDM16*, *TMEM26*, and *CD137*) emerges in response to irisin expression, and protein levels of UCP1 increase in subcutaneous white adipose tissue (Li et al. 2019). Such transformation of white adipose tissue might be used to treat metabolic disorders. The possibility of *Ucp1* activation in white adipose tissue, including the use of irisin, is considered as one of the strategies to combat obesity and thus to fight against T2D and cardiovascular disorders. Two cell-specific transcriptional coactivators, PPARγ coactivator 1α (PGC-1α, encoded by *Ppargc1a*) and PR domain-containing 16 (PRDM16), were described as major molecular determinants of the brown adipose phenotype (Kajimura et al. 2009). Generation of *Ppargc1a*-null mice revealed that PGC-1α is essential for expression of *Ucp1*, but not for brown fat cell differentiation (Lin et al. 2004; Uldry et al. 2006). Cell-based studies showed that, upon forskolin treatment (which imitates noradrenergic stimulation), PRDM16 could be recruited to the enhancer of *Ucp1* to induce its expression in undifferentiated murine embryonic fibroblasts. PRDM16 was identified as a universal brown fat cell-fate determination factor (Seale et al. 2007) that promotes differentiation of Myf5-positive myogenic precursor cells into brown adipocytes while banning myogenic differentiation (Seale et al. 2008). PRDM16 was additionally shown to induce acute expression of BAT-specific genes such as *Ucp1* and *Ppargc1a*, when ectopically expressed in murine embryonic fibroblasts (Seale et al. 2007) and to play a role in BAT-specific gene expression in beige fat cells (Cohen et al. 2014; Seale et al. 2011). The molecular mechanism of transcriptional activation by PRDM16 is still unclear (Iida et al. 2015). Recent studies showed that early B-cell factors (EBF) are required for chronic cold-induced BAT recruitment. Both EBF1 and EBF2 cooperate with estrogen-related receptor α and PGC-1 α to promote *Ucp1* transcription (Angueira et al. 2020).

### Uncoupling protein 1

UCP1 is a transmembrane protein carrier with a molecular weight of 32 kDa and, like all members of the UCP family, is located in the inner membrane of mitochondria (Hoang et al. 2013). UCP1 allows protons from the intermembrane space to penetrate back into the mitochondrial matrix, bypassing the oxidative phosphorylation of ADP. As a result, ATP synthesis is reduced and free cell respiration is intensified to release energy in the form of heat. Despite the existence of several homologues, UCP1 is the only member of the family that performs a true thermogenic function (Ricquier 2011). UCP1 constitutes about 10% of the brown adipocyte mitochondrial membrane protein, in contrast to UCP2 и UCP3, the content of which does not exceed 0.1% (Kozak and Anunciado-Koza 2009).

UCP1 was found in BAT in the 1970s and was sequenced later. It was believed earlier that *Ucp1* is only expressed in newborns, but both the UCP1 protein and its mRNA were found in adults more recently (Bouillaud et al. 1988; Lean et al. 1986). Brown fat cells most intensely express *Ucp1* in the human body, but the *Ucp1* mRNA becomes detectable in subcutaneous white fat in response to certain stimuli, such as exercise training (Norheim et al. 2014; Otero-Díaz et al. 2018) and cold exposure (Barbatelli et al. 2010; Finlin et al. 2018; Kern et al. 2014), and is found in skeletal muscle of mice (Gorski et al. 2018).

Thermogenesis in BAT is directly controlled by the endings of sympathetic nerve fibers, which are abundant in the tissue. Norepinephrine released by sympathetic nerve fibers activates brown adipose hormone-sensitive lipase, which hydrolyzes intracellular triglycerides through a cascade of reactions. UCP1 is activated by the resulting free fatty acids, and the phosphorylation-uncoupling process intensifies (Fedorenko et al. 2013).

Free radicals are generated when electrons are transferred to oxygen molecules during oxidative phosphorylation. However, these toxic compounds are balanced with present antioxidants in normal conditions. When this balance is disrupted, oxidative stress occurs to induce a number of pathophysiological conditions, including T2D (Brondani et al. 2012). By reducing the membrane potential, UCP1 and its homologues reduce the generation of free radicals and thus protect the cell against oxidative stress (Brand et al. 2004).

Despite the fact that most members of the UCP family are capable of reducing the proton gradient, the true thermogenic function is only performed by UCP1. Thus, UCP1 is one of the main focuses of research into the molecular biological processes associated with energy metabolism and its disorders. A significant part of *Ucp1* studies aimed at detecting its single nucleotide polymorphisms (SNPs) and analysing their phenotypic manifestations.

Here we review the data on the most significant SNPs of the *Ucp* family genes and their association with cardiometabolic diseases (CMDs) and CMD risk factors.

### Role of *Ucp1* polymorphisms in cardiometabolic diseases

**Fig. 1:**
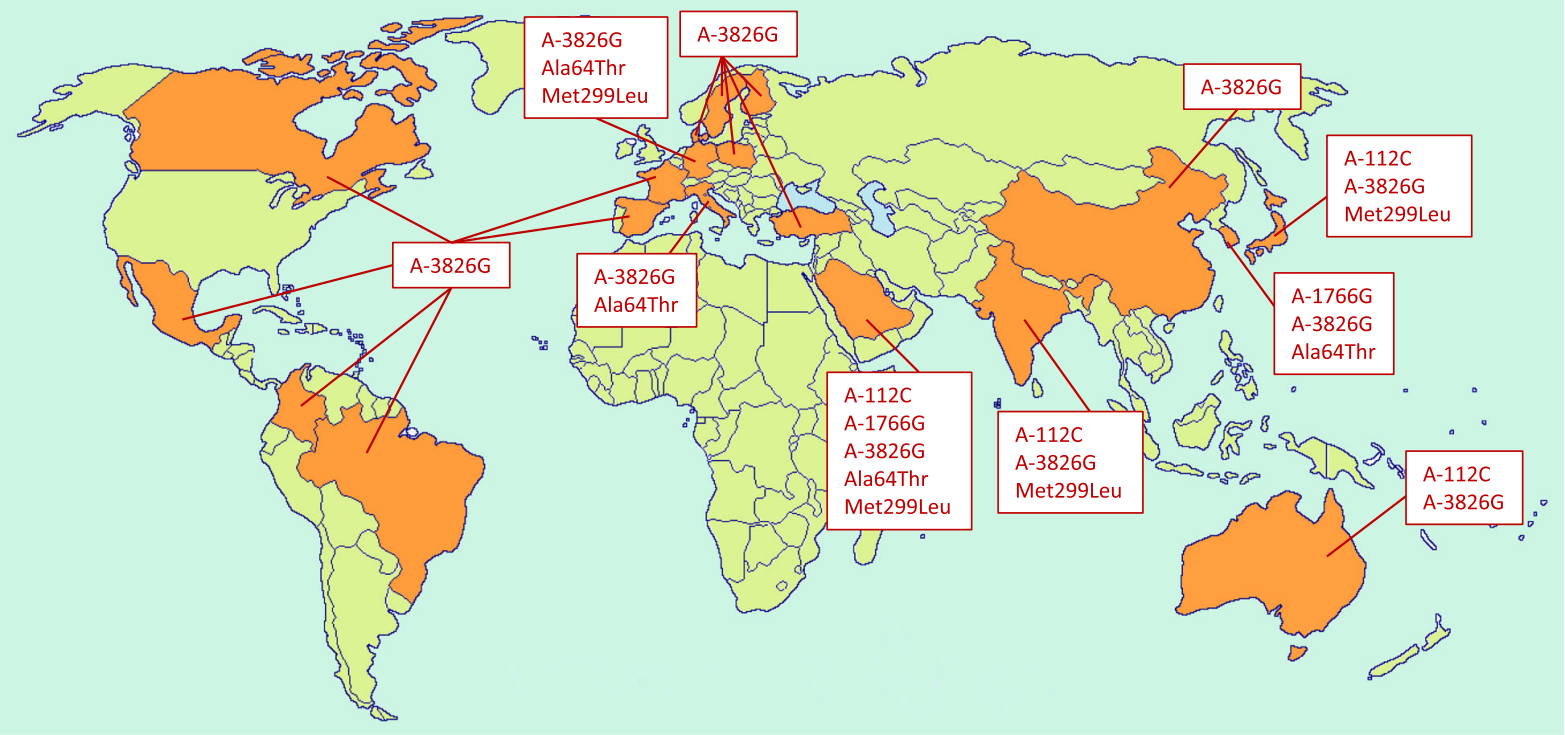
World map showing the investigation of the association of *Ucp1* polymorphisms with CMDs or CMD risk factors

CMDs are chronic diseases that develop latently throughout life and progress to an advanced stage by the time symptoms appear. CMDs are lifestyle dependent and include a set of risk factors for the development of chronic pathological conditions with adverse effects on the cardiovascular function, such as atherosclerosis, arterial thrombosis, and metabolic disorders leading to T2D and related complications (Flouris et al. 2017; Scottish Intercollegiate Guidelines Network (SIGN) 2017). Many CMDs have a multifactorial origin; i.e., both genetic and environmental factors are involved in their development. Numerous studies showed that an increased BMI, insulin secretion, and increased blood pressure may be due to a genetic predisposition. Mutations in the *Ucp* genes can increase the risk of obesity and associated metabolic disorders (Brondani et al. 2014a). The *Ucp1* gene was assigned to the long arm of chromosome 4 in q31.1. *Ucp1* is split into six exons, spans about 13 kb, and contains a 9-kb transcribed region (Cassard et al. 1990). A large number of studies (Table 1) described SNPs in the noncoding region of the *Ucp1* gene, such as A–3826G (rs1800592), A–1766G (rs3811791), and A–112C (rs10011540), as well as in its coding part, which includes Ala64Thr (rs45539933) and Met229Leu (rs2270565) (Fig. 1). However, the influence of *Ucp1* genetic variants was studied poorly, if at all, in Russia and Eastern Europe, where the CMD prevalence rates remain high (Flouris et al. 2017) (Fig. 1).

**Table 1:** Studies on the association between the *Ucp1* polymorphisms and CMDs or CMD risk factors in different populations

SNP(s)	Population and participants	Association		Allele/genotype frequencies	Reference
		+/−	Condition		
A–3826G	Australian: overweight/obese F = 526	+	↑ BMI (*P* = 0.02), ↑ insulin (*P* = 0.03, not adjusted), and ↑ fasting glucose concentrations (*P* = 0.01, adjusted for BMI)	G allele: 0.23	(Heilbronn et al. 2000)
A–3826G	Brazil: T2D patients *N* = 981 and controls *N* = 534	–	T2D (*P* > 0.05)	AA: 49.9%, AG: 37.7%, GG: 12.4%, G allele: 0.313 in diabetics; AA: 49.3%, AG: 39.5%, GG: 11.2%, G allele: 0.310 in controls	(de Souza et al. 2013)
A–3826G	Canadian: parents *N* = 123 and offsprings *N* = 138 from 64 families	–	Body fat, RMR, and absolute changes in body fat over a 12-year period	G allele: 0.28	(Oppert et al. 1994)
		+	↑ body fat gain over time (*P* < 0.05)		
A–3826G	Chinese: T2D patients *N* = 792, including DR group: PDR *N* = 220 and NPDR *N* = 228; DNR group: *N* = 334	+	↑ risk of PDR (additive model OR = 1.72, 95% CI: 1.06–2.79, *P* = 0.03)	AA: 20.7%, AG: 49.3%, GG: 30.0%, G allele: 54.6% in PDR group; AA: 28.1%, AG: 48.2%, GG: 23.7%, G allele: 47.8% in DNR group	(Zhang et al. 2015)
		+	PDR (OR = 1.32, 95% CI: 1.03–1.68, *P* = 0.03)		
		–	DR and DNR or NPDR and DNR		
A–3826G	Chinese: hypertensive subjects M = 573 and F = 589; normotensive subjects M = 373 and F = 672	–	EH	AA: 25.12%, AG: 50.85%, GG: 24.06%, G allele: 49.47% in normotensives; AA: 25.22%, AG: 52.01%, GG: 22.77%, G allele: 48.78% in hypertensives	(Sun et al. 2018)
A–3826G	Chinese: T2D patients *N* = 3107, including DR = 662	–	DR (OR = 1.001, *P* = 0.993)	AA (n): 2578, AC (n): 469, CC (n): 14	(Jin et al. 2020)
A–1766G		–	DR (OR = 0.949, *P* = 0.488)		
A–112C		+	DR (OR = 1.368, *P* = 0.004)		
A–1766G	Chinese: T2D patients *N* = 929, nondiabetic controls *N* = 1044	+	↑ risk of T2D (OR = 1.42, *P* = 0.042), ↑ level of TG (β = 0.048, *P* = 0.034) under recessive model	AA: 53.8%, AG: 37.3%, GG: 8.9%, G allele: 27.6% in T2D patients; AA: 53%, AG: 40.6%, GG: 6.4%, G allele: 26.7% in controls	(Dong et al. 2020)
A–3826G	Colombian: T2D cases M = 190 and F = 355; controls M = 126 and F = 323	+	A allele and T2D (OR = 0.78; 95% CI: 0.63–0.97; *P* = 0.02)	A allele: 0.54 in T2D cases and 0.60 in controls	(Franco-Hincapié et al. 2014)
A–3826G	Danes:young healthy subjects M = 177 and F = 176	–	BMI, fat mass, or weight gain during childhood and adolescence	G allele: 25.3% (95% CI: 22.2–28.4%)	(Urhammer et al. 1997)
Ala64Thr	Danes: males with juvenile obesity *N* = 156; controls *N* = 205: lean controls *N* = 79	–	Obesity and weight gain during childhood or adolescence, or insulin sensitivity	64Thr allele: 8.2% in juvenile obese subjects, 8.8% in controls, and 8.2% in lean controls	
Met229Leu		–		229Leu allele: 8.2% in obese subjects, 8.1% in controls, and 5.6% in lean controls	
A–3826G	Finnish: T2D patients M = 38 and F = 32; controls M = 55 and F = 68	–	T2D, body weight or BMI in diabetics or controls	G allele: 38.6% in diabetics, 34.1% in controls	(Sivenius et al. 2000)
A–3826G	Finnish: obese premenopausal women *N* = 77	–	Weight gain after a VLCD	G allele: 0.19	(Fogelholm et al. 1998)
A–3826G	French: overweight patients *N* = 163	+	↓ weight loss after low calorie diet (*P* < 0.05)	G allele: 0.27	(Fumeron et al. 1996)
A–3826G	German: T2D patients *N* = 517	–	Neuropathy (*P* = 0.79), retinopathy (*P* = 0.48), and nephropathy (*P* = 0.93)	AA: 49.9%, AG: 45.6%, GG: 4.5%, G allele: 0.27	(Rudofsky et al. 2007)
Ala64Thr	German children and adolescents: obese subjects *N* = 293, F = 53%; lean subjects *N* = 134, F = 46%	–	Early-onset obesity	64Thr allele: 8.2% in obese, 4.1% in lean individuals	(Hamann et al. 1998)
Met229Leu		–		229Leu allele: 10.4% in obese, 12.0% in lean individuals	
A–3826G	Indian: M = 49 and F = 47	+	GG genotype and BMI (*P* < 0.01), SBP (*P* < 0.01) and DBP (*P* < 0.05) in females	GG:13.5%, AG: 46.5%, AA: 39.9%	(Dhall et al. 2012)
A–3826G	Indian: T2D subjects M = 353 and F = 457; normal glucose-tolerant subjects M = 374 and F = 616	–	T2D	AA: 36%, AG: 46%, GG: 18% G allele: 0.41 in T2D subjects. AA: 40%, AG: 45%, GG: 15% G allele: 0.38 in normal glucose-tolerant subjects	(Vimaleswaran et al. 2009)
A–112C		–		AA: 63%, AC: 33%, CC: 4%, C allele: 0.21 in T2D subjects. AA: 62%, AC: 34%, CC: 4%, C allele: 0.21 in normal glucose- tolerant subjects	
Met299Leu		–		MetMet: 76%, MetLeu: 23%, LeuLeu: 1%, 299Leu allele: 0.12 in T2D subjects; MetMet: 73%, MetLeu: 26%, LeuLeu: 1%, 299Leu allele: 0.14 in normal glucose-tolerant subjects	
A–3826G	Italian: severely obese nondiabetic individuals M = 40 and F = 72	+	IR in morbid obesity	AA: 25.9%, AG + GG: 74.1% in total obese population; AG + GG: 88% in IR (+) and 63% in IR (−) (OR = 4.3, 95% CI: 1.6–11.7; *P* = 0.003)	(Bracale et al. 2012)
A–3826G	Italian: T2D individuals M = 56.6%; controls M = 41.2%	–	T2D (OR = 0.85, 95% CI: 0.65–1.11; *P* = 0.221)	G allele: 0.24	(Montesanto et al. 2018)
		+	Risk of nephropathy (OR = 0.57, 95% CI: 0.33–0.98; *P* = 0.031)		
		–	Ischemic heart disease and stroke (OR = 1.10, 95% CI: 0.74–1.64; *P* = 0.643)		
Ala64Thr		–	T2D (OR = 0.99, 95% CI: 0.61–1.6; *P* = 0.969)	64Thr allele: 0.063	
		+	Risk of retinopathy (OR = 0.31, 95% CI: 0.12–0.82; *P* = 0.010)		
		–	Ischemic heart disease and stroke (OR = 1.08, 95% CI: 0.52–2.26; *P* = 0.837)		
A–3826G	Japanese: individuals sampled during cold *N* = 1080 and hot season *N* = 979	+	VFA during cold season (*P* = 0.0197)	G allele: 0.51	(Nakayama et al. 2013)
A–3826G	Japanese: subjects without a history of cardiovascular disease M = 231 and F = 347	+	GG genotype and HT in males (OR = 2.32, 95% CI: 1.08–4.99) and older subjects (OR = 1.89, 95% CI: 1.00–3.57)	AA: 24.0%, AG: 50.0%, GG: 26.0%, G allele: 0.51	(Kotani et al. 2007)
A–112C	Japanese: T2D patients M = 180 and F = 140, controls M = 145 and F = 105	+	T2D (*P* = 0.017)	С allele: 6.2% in healthy controls, 10.2% in T2D patients	(Mori et al. 2001)
Met299Leu		+	T2D (*P* = 0.038)	229Leu allele: 7.2% in healthy controls, 10.8% in T2D patients	
A–3826G		–	T2D	G allele: 53.6% in healthy controls, 49.7% in T2D patients	
A–112C	Japanese: T2D cases M = 55 and F = 38	+	↑ FIRI (*P* = 0.0085), HOMA-IR (*P* = 0.0089), and HLC (*P* = 0.012)	AA: 88.2%, AC: 10.7%, CC:1.1%	(Fukuyama et al. 2006)
A–3826G		–	T2D	AA: 32.3%, AG: 48.4%, GG: 19.3%	
A–3826G	Japanese: obese F = 113, healthy non-obese F = 76	+	GG genotype and resistance to weight loss among obese (*P* < 0.05)	G allele: 0.46 in obese and 0.45 in healthy non-obese	(Kogure et al. 1998)
A–3826G	Japanese: healthy postmenopausal group F = 182 and premenopausal group F = 99	+	↑ body weight (*P* = 0.048) in premenopausal women and changes in serum TG (*P* = 0.049) and HDL (*P* = 0.020) levels in postmenopausal women	AA: 23.7%, AG: 53.2%, GG: 23.1%, G allele: 0.50	(Matsushita et al. 2003)
A–3826G	Japanese: healthy boys *N* = 22	+	GG genotype and ↓ TEM to fat intake (*P* < 0.05)	AA+AG (n): 13, GG (n): 9	(Nagai et al. 2003)
A–3826G	Japanese: healthy children *N* = 19	+	GG genotype and ↓ cold-induced thermogenesis (*P* < 0.05)	AA+AG (n): 14, GG (n): 5	(Nagai et al. 2007)
A–3826G	Japanese: M = 251	+	AG genotype and BMI (*P* = 0.016)	AA: 24.3%, AG: 48.2%, GG: 27.5%	(Nakano et al. 2006)
A–3826G	Korean: obese patients M = 44 and F = 146	+	AG/GG genotypes and ↑ DBP (*P* = 0.023) and LDL-C (*P* = 0.011); GG genotype and ↓ HDL-C (*P* < 0.05) and ↑ atherogenic index	AA: 22.1%, AG: 53.7%, GG: 24.2%, G allele: 0.51	(Oh et al. 2004)
A–3826G	Korean: F = 387	+	[−3826G/−1766G] Ht and ↑ body fat percent (*P* = 0.045)	NA	(Kim et al. 2005)
A–1766G		+	AG/GG genotypes and abdominal subcutaneous fat (*P* = 0.015), abdominal visceral fat (*P* = 0.013), ↑ WHR (*P* = 0.008), body fat mass (*P* = 0.023), and percent body fat (*P* = 0.014)	AA:57.4%, AG: 37.7%, GG: 4.9%, G allele: 0.238	
A–3826G A–1766G Ala64Thr	Korean: overweight F = 453	+	[−3826G/ –1766A/64Thr] Ht and ↓ abdominal fat tissue area (*P* = 0.02), fat tissue area at thigh (*P* = 0.008), body fat mass (*P* = 0.002), and WHR (*P* = 0.01) [−3826G/ –1766A/64Ala] Ht and reduction of WHR and body fat mass by VLCD (*P* = 0.05–0.006)	NA	(Shin et al. 2005)
A–3826G	Mexican adolescents: normal weight *N* = 159 and overweight/obese *N* = 111	+	↑ percentage of fat (*P* = 0.002) and muscle weight (*P* = 0.019) in a recessive model	AA: 16.2%, AG: 55.9%, GG: 27.9% in obese; AA: 17%, AG: 50.9%, GG: 32.1% in controls	(Sámano et al. 2018)
A–3826G	Mixed ethnicity population: obese patients *N* = 150, F = 80%	+	↓ values of weight, body fat and free fat mass (*P* < 0.05); GG genotype and ↓ tendency of T2D	AA: 41.3%, AG: 45.3%, GG: 13.4%, G allele: 0.36	(Nicoletti et al. 2016)
A–3826G	Polish: overweight/obese individuals M = 38 and F = 80	–	BMI	AA: 51.38%, AG: 33.94%, GG:14.68%; G allele: 30.5%.	(Kieć-Wilk et al. 2002)
		+	↑ fasting levels of TG (*P* < 0.04) and ↓ HDL-C (*P* = 0.004)		
A–3826G	Polish: embers of obese Caucasian families M = 38 and F = 84	–	Susceptibility to obesity and glucose tolerance parameters	NA	(Malczewska-Malec et al. 2004)
A–112C	Saudi Arabian: obese patients M = 138 and F = 199; healthy volunteers M = 76 and F = 79	–	Obesity	A allele/C allele (n/n): 627/47 in obese patients, 283/27 in controls	(Chathoth et al. 2018)
A–1766G		+	A allele and moderate obesity (OR = 2.89, 95% CI 1.33–6.25; *P* = 0.007)	A allele/G allele (n/n): 624/50 in obese patients, 298/12 in controls	
A–3826G		+	Obesity after adjusting age, sex, and T2D (OR = 1.52, 95% CI: 1.10–2.08; *P* = 0.009)	A allele/G allele (n/n): 443/231 in obese patients, 227/83 in controls	
A–3826G	Spanish: obese individuals *N* = 159, normal weight, *N* = 154	+	↑ BMI (*P* = 0.03), ↑ percentage of body fat (*P* < 0.04), ↑ SBP (*P* = 0.009), ↑ DBP (*P* = 0.02) in obese group	G allele: 0.21 in healthy, 0.19 in obese individuals (*P* = 0.574)	(Forga et al. 2003)
A–3826G	Spanish: obese M = 38 and F = 55; non-obese M = 122 and F = 117	+	Obesity in women (*P* = 0.019)	G allele: 0.3 in obese (0.28 in males and 0.31 in females), 0.24 in non-obese (0.32 in males and 0.17 in females) (*P* = 0.008)	(Ramis et al. 2004)
A–3826G	Swedish: obese subjects M = 310 and F = 364, non-obese subjects M = 54 and F = 257	–	Obesity-related phenotypes and weight gain	G allele: 0.25 in obese, 0.24 in non-obese subjects	(Gagnon et al. 1998)
A–3826G	Turkish:obese M = 83 and F = 63; lean individuals M = 77 and F = 17	+	↑cholesterol levels	G allele: 0.30 in obese, 0.31 in lean individuals	(Proenza et al. 2000)
A–3826G	Turkish children and adolescents: obese *N* = 268 and non-obese = 185	+	GG denotype and obesity (OR = 2.02, 95% CI 1.17–3.47; *P* = 0.010),↑ TG levels in obese subjects (*P* = 0.048), ↓ HDL-C (*P* = 0.017)	AA: 46.3%, AG: 33.2%, GG: 20.5%, G allele: 37.1% in obese; AA: 46.5%, AG: 42.2%, GG: 11.4%, G allele: 32.4% in controls	(Gul et al. 2017)
Ala64Thr	White subjects: obese *N* = 93 and non-obese *N* = 69	–	BMI or obesity	64Thr allele: 12.0%	(Herrmann et al. 2003)
		+	WHR (*P* = 0.003) after adjustment for gender, age, BMI, and T2D		

*Abbreviations*: *F* Females, *M* Males, *BMI* Body mass index, *RMR* Resting metabolic rate, *DR* Diabetic retinopathy, *PDR* Prolifirative diabetic retinopathy, *NPDR* Non-prolifirative diabetic retinopathy, *DNR* Diabetes without retinopathy, *EH* Essential hypertension, *T2D* Type 2 diabetes, *VLCD* Very low calorie diet, *IR* Insulin resistance, *VFA* Visceral fat accumulation, *HT* Hypertension, *FIRI* Fasting insulin resistance index, *HOMA-IR* Homoeostasis model assessment of insulin resistance, *HLC* Hepatic lipid content, *HDL-C* High density lipoprotein cholesterol, *TEM* Thermic effect of a meal, *Ht* Haplotype, *SBP* Systolic blood pressure, *DPB* Diastolic blood pressure, *LDL-C* Low density lipoprotein cholesterol, *WHR* Waist-to-hip ratio, *TG* Trygliceride, *NA* Not available

### A–3826G polymorphism of the *Ucp1* gene

**Fig. 2:**
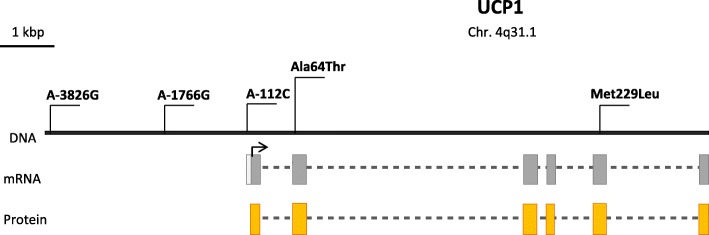
Schematic structure of the *Ucp1* gene and its best-studied polymorphisms

The A/G SNP at position − 3826 bp (rs1800592) of the 5′ noncoding region of the gene (Fig. 2) was the focus of initial publicationsin the field. The SNP was found by Canadian scientists in 1994 in a study of the genetic characteristics and family histories of patients with elevated BMIs. The study was the first to associate the G allele with a higher BMI, the risk of T2D, and lipid metabolism disorders (Oppert et al. 1994).

It was later established that the SNP is in the regulatory region of the gene and that obese G allele carriers have reduced *Ucp1* mRNA expression (Esterbauer et al. 1998), suggesting functional significance for the SNP. An enhancer sequence with five hexameric binding sites for nuclear retinoic acid receptors—RARα, RARβ, retinoid X receptors (RXR) and peroxisome proliferator-activated receptors (PPARγ)—was found in close proximity to the A–3826G SNP (del Mar Gonzalez-Barroso et al. 2002). Polymorphisms of this region are likely to disrupt the transcription factor binding sites, thus reducing *Ucp1* expression of to some extent.

A statistically significant correlation between A–3826G and phenotypic characteristics was observed in numerous studies of the BMI, body fat content, and weight loss or gain intensity, the AA genotype being associated with the lowest risk in all cases (Brondani et al. 2012; Forga et al. 2003). However, the contribution of the mutant allele to the pathogenesis of diseases was not confirmed in some studies of A–3826G in patients with metabolic disorders and cardiovascular diseases from different populations (Gagnon et al. 1998; Kieć-Wilk et al. 2002; Schäffler et al. 1999; Urhammer et al. 1997).

Japanese researchers showed that a decrease in body weight in obese G allele carriers was lower than in A allele carriers, with similar low-calorie diet and exercise regimen (4.3 ± 2.6 vs. 7.4 ± 4.2 kg; *p* < 0.05) (Kogure et al. 1998). Similar results were obtained by scientists from France: the GG genotype determined a resistance to low calorie diet (*P* < 0.05) (Fumeron et al. 1996), and Finland (Fogelholm et al. 1998), but the results did not reach statistical significance. In addition, G allele carriers with the genomic existence of the Trp64Arg substitution in the beta 3-adrenergic receptor gene were more susceptible to weight gain than A allele carriers in the Finnish population (Sivenius et al. 2000).

The G allele was associated with an increased BMI (*P* = 0.02) and a higher blood glucose concentration in the Australian female population (*P* = 0.01, adjusted for BMI), and its higher frequency was observed in women with T2D (*P* = 0.06, adjusted for BMI) (Heilbronn et al. 2000). In the work by Spanish authors, the G allele was more common in men, but the association with overweight was revealed only in women: the G allele frequency in obese female patients was higher than in non-obese female subjects (0.31 vs. 0.17, *P* = 0.008) (Ramis et al. 2004). The association of the GG genotype with obesity and a higher blood pressure was also seen among Indian females but not among males (Dhall et al. 2012).

In contrast, in Japan, the nucleotide substitution was not associated with hypertension in women, while susceptibility to the disease in the male population was determined by the GG genotype togetger with the age and increased BMI (Kotani et al. 2007). In addition, male G allele carriers had higher BMIs as compared with the male carries of the AA genotype. However, the highest BMI values were observed in individuals with the AG genotype among young Japanese males (*P* = 0.016) (Nakano et al. 2006). In the Italian population, the G allele variant was associated with the development of insulin resistance in obese patients (Bracale et al. 2012), which increases the risk for diabetes. In northwestern Colombia, the A allele negatively correlated with T2D and, according to the authors, could act as a protective factor in the population studied (Franco-Hincapié et al. 2014). In Saudi Arabia, a statistically significant association was found for the minor G allele and an increased BMI (Chathoth et al. 2018). In Mexican adolescents, the –3826G variant was associated with higher percentage of fat (*P* = 0.002) and muscle mass (*P* = 0.019) in the body, but no differences in allele frequencies was found between corpulent and lean students (Sámano et al. 2018). Opposite results were obtained by Brazilian researchers. In a sample of patients with BMI ≥ 35 kg/m^2^, the G allele was associated with lower weight and body fat content and GG genotype carriers were less likely to be diagnosed with T2D as compared with carriers of the wild-type A allele (Nicoletti et al. 2016).

Proenza et al. observed increases in total cholesterol levels associated with a high BMI and low high density lipoprotein cholesterol (HDL-C) in GG carriers that among 271 patients of Turkish origin (Proenza et al. 2000). The G allele was more frequent in patients with triglyceridemia as compared with subjects without it (42.9 vs. 34.4%, *P* = 0.048) among obese children and adolescents (Gul et al. 2017). In a study of obese families from southern Poland, the GG genotype was similarly associated with decreased HDL-C levels and higher levels of triglycerides and free fatty acids as compared with the AG and AA genotypes (Kieć-Wilk et al. 2002). Similar results were obtained for HDL-C levels in a group of Japanese postmenopausal women (*P* = 0.020) (Matsushita et al. 2003) and obese patients from the Korean population. In addition, Korean G allele carriers showed an increased diastolic blood pressure and higher low-density liprotein cholesterol (LDL-C) levels (Oh et al. 2004). In studies conducted in East Asians, the atherogenic index, which reflects the LDL-C/HDL-C ratio in the blood, was higher in GG genotype carriers. A high LDL-C content and an elevated atherogenic index increase the risk of atherosclerosis and cardiovascular disease (Jia et al. 2010).

The A–3826G SNP is associated with the development of microvascular complications in diabetes mellitus. In the Chinese population, the G allele was associated with an increased risk of proliferative diabetic retinopathy in patients with T2D (Zhang et al. 2015). However, no association of the SNP with microvascular complications of diabetes was found in a study involving a European sample (Rudofsky et al. 2007). On the contrary, the G allele was recently reported to act as a protective factor against the development of retinopathy in residents of central Italy suffering from T2D (Montesanto et al. 2018).

No significant association with overweight or diabetes was found in studies of the A–3826G polymorphism in the Danish (Urhammer et al. 1997), Swedish (Gagnon et al. 1998; Mottagui-Tabar et al. 2007), German (Schäffler et al. 1999), and Polish (Kieć-Wilk et al. 2002; Malczewska-Malec et al. 2004) populations.

Mutations in the *Ucp1* gene can impare the main BAT function, that is, nonshivering thermogenesis. Healthy children with the GG genotype showed a lowere capacity of the thermic effect of a high fat meal (*P* < 0.05) (Nagai et al. 2003) and lower capacity for thermogenesis after exposure to cold (Nagai et al. 2007) as compared with AA and AG genotype carriers in the Japanese population. Accumulation of visceral fat, which is one of the main risk factors for cardiovascular diseases, may also be associated with a decrease in BAT activity. Nakayama et al. studied associations of the A–3826G polymorphism with visceral fat accumulation during cold and hot seasons. An association of the G allele with increased visceral fat (*P* = 0.0198) was only observed in the cold months of the year (Nakayama et al. 2013). Such observations confirm the fact that BAT becomes metabolically more active in adults after cold exposure and is detected in winter more frequently than in summer in humans (Saito et al. 2009). The A/G substitution is in the regulatory region of the gene and may decrease *Ucp1* transcription, thus impairing nonshivering thermogenesis. G allele carriers are consequently more prone to visceral obesity in winter. Nonshivering thermogenesis merely contributes to energy expenditure during the hot season, and no association between the mutation and the amount of fat in the abdominal cavity was therefore detected in summer (Nakayama et al. 2013).

Inconsistent results of *Ucp1* A–3826G studies may partly be explained by the differences in climatic conditions in which they were carried out. As mentioned above, there is evidence that active BAT is more frequently observed in cold seasons in humans (van Marken Lichtenbelt et al. 2009; Saito et al. 2009), yet the season was not taken into consideration in most of the association studies of *Ucp1* polymorphisms. There is no doubt that weather and climate play a major role in human health. A worldwide population analysis showed increased rates of coronary events during increased cold periods in warm climates (Barnett et al. 2005). Populations of the coldest regions (Northern Sweden, North Karelia, and Kuopio) demonstrated little changes in coronary event rates with changes in temperatures (Barnett et al. 2005). This trend was supported by findings from a study in Siberia, where mortality from ischaemic heart disease did not rise with the decreasing temperature. In contrast, death rates rose as temperature fell below 18 °C in western Europe. This could be explained in part by genetic and lifetime adaptations to cold (Donaldson et al. 1998).

Ethnic differences in the allele frequencies of the polymorphism are also important to consider. A population analysis showed that the A allele is more frequent at high latitudes and that its frequency inversely correlates with the level of solar radiation. Of 52 populations studied, the lowest frequency of the A allele was observed in Africa (Hancock et al. 2011). Thus, environmental factors (climate, geographical location, dietary habbits, and daily activity) could modify the association between the *Ucp1* polymorphisms and CMDs and contribute to the differences in association (Flouris et al. 2017). Moreover, the substitutions may alter the genotypic response to environmental influences by activating different molecular factors and pathways, thus modifying the risk for the disease (Slavich and Cole 2013).

A variation in haplotypes containing the A–3826G polymorphism and other SNPs of the *Ucp* genes can also affect the study results. Shin et al. studied three haplotypes containing the –3826G allelic variant in a group of Korean obese women. Both positive and negative correlations with obesity were observed with different haplotypes (Shin et al. 2005). It is interesting to note that the –3826G allele contained in different haplotypes with *Ucp1*, *Ucp2* and *Ucp3* polymorphisms reduced the risk of hypertension, while the A allele increased the probability of disease development in a Chinese population (Sun et al. 2018).

### A–1766G polymorphism of the *Ucp1* gene

The substitution of A for G at nucleotide position − 1766 (rs3811791) is another polymorphism in the upstream region of the *Ucp1* gene (Fig. 2). The SNP was first discovered by Korean researchers in 2004. The SNP is upstream of the promoter region of the gene and is about 2 kb closer to the transcription start site than the A–3826G SNP described earlier (Kim et al. 2005). The –1766G allelic variant was associated with several obesity parameters, such as the waist–hip ratio (WHR) (*P* = 0.008), body fat mass (*P* = 0.023), body fat percentage (*P* = 0.014), and abdominal fat amount in the same study. The interaction of the A–3826G and A–1766G polymorphisms was additionally considered in the Korean population. Patients with the haplotype [−3826G/−1766G] had the highest body fat percentage, while the haplotype [−3826G/−1766A] was associated with lower body fat (Kim et al. 2005). The data from the study were consistent with the results obtained by Shin et al. The –1766G allele showed a significant association with high values of the WHR, fat mass, body fat percentage, and abdominal fat amount in the Korean population (Shin et al. 2005). In Saudi Arabia, the association of the A–1766G substitution with obesity was detected after dividing the sample by BMI into subgroups with grade II and III obesity. A significant increase in –1766G allele frequency was observed in the subgroup with grade II obesity (BMI = 35–39.9) as compared with a control cohort [OR = 2.89, 95% CI 1.33–6.25; *P* = 0.007] (Chathoth et al. 2018). In a recent study conducted in China, the polymorphism was associated with a significantly higher risk of T2D (adjusted OR = 1.42, 95% CI: 1.01–1.99, *P* = 0.042) and higher TG levels (β = 0.048, *P* = 0.034) under the recessive model (Dong et al. 2020).

### A–112C polymorphism of the *Ucp1* gene

Phenotypic manifestations were much less studied for the A–112C polymorphism (rs10011540) (Table 1). It is known that the A–112C polymorphism affects the consensus motif of the (T(G/A)TTT(T/G)(G/T)) insulin response element (IRS). The mutant С allele reduced the activity of the *Ucp1* gene promoter in in vitro experiments, confirming the involvement of the IRS in regulating transcription (Mori et al. 2001). Insulin was shown to induce *Ucp1* expression (Teruel et al. 1998; Valverde et al. 2003). It is therefore possible to assume that the A–112C substitution weakens IRS affinity for transcription factors, thus reducing the activity of the *Ucp1* promoter.

There are data on the association of the A–112C polymorphism with insulin resistance parameters in T2D patients. The C allele was more frequent in patients with T2D than in a control group in the Japanese population (*P* = 0.017) (Mori et al. 2001). Another group of Japanese researchers showed that, among T2D patients, carriers of the –112C allelic variant had higher levels of fasting insulin resistance index (FIRI) (*P* = 0.0085), homeostasis model assessment (HOMA) index (*P* = 0.0089), and hepatic lipid content (HLC) (*P* = 0.012). In addition, the –112C allele was in linkage disequilibrium with the –3826G variant. However, no statistically significant association was detected between –112C and BMI or visceral fat content (Fukuyama et al. 2006).

In the Indian population, CC genotype carriers had a significantly higher systolic blood pressure as compared with carriers of the AA genotype (126 ± 20 mmHg vs. 119 ± 17 mmHg, *P* = 0.05). The A–112C polymorphism was not associated with T2D in the study, but acted as part of the haplotype [−3826A/−112C/229Met] to increase the risk of T2D in Indians (Vimaleswaran et al. 2009). In a Chinese population, the –112C allelic variant was identified as a risk factor for diabetic retinopathy (OR = 1.368 *P* = 0.004) (Jin et al. 2020).

### Polymorphisms of the coding region of the *Ucp1* gene

A number of polymorphisms in exons of the gene can also be attributed to risk factors for cardiovascular diseases and metabolic disorders (Table 1). The SNP rs45539933 is a substitution of G for A at nucleotide position + 1068 and results in a substitution of Ala for Thr at amino acid position 64 (Fig. 2). The SNP was detected in exon 2 of the *Ucp1* gene by Hamann et al. The frequency of the mutant allele in a group of obese adults and children was two times higher than in a control group in the German population (8.2 vs. 4.1%, nominal *P* = 0.029, corrected *P* = 0.058) (Hamann et al. 1998). The Ala64Thr polymorphism was associated with the WHR in Caucasians. Carriers of the 64Thr allele had higher waist circumference values and, consequently, higher WHRs (*P* = 0.003) (Herrmann et al. 2003). On the contrary, the 64Thr allele was associated with lower fatness, lower WHR, and lower fat mass in a study involving Korean female patients (Shin et al. 2005). Carriage of the 64Thr allele reduced the risk of retinopathy in T2D patients from the Italian population (Montesanto et al. 2018).

It is difficult to provide a clear explanation for the discrepancies observed in the results at the moment. Ethnic differences in SNP allele frequencies are necessary to take into account when studying different populations; e.g., the 64Thr allele in Koreans was less frequent than in Caucasians. Gender differences between samples are also of importance; e.g., only women were included in the study by Korean authors. Because obesity and diabetes are multifactorial in nature, the diet can distort the results of studies on the association between polymorphisms and obesity (Luan et al. 2001). Different dietary patterns of Caucasians and Asians might contribute to the inconsistency of research results.

Hamann et.al. detected three other point mutations in the coding region of the *Ucp1* gene: Arg40Trp (rs573078239) in exon 1 and Lys257Arg (rs1431343082) and Met229Leu (rs2270565) in exon 5, but none of them showed an association with the development of obesity (Hamann et al. 1998). The Ala64Thr and Met229Leu substitutions did not correlate with BMI in Caucasians of Danish ancestry. The mutant alleles 64Thr and 229Leu are found in the amino acid sequences of UCP1 in rodents (hamsters and rats) and rabbits, whose BAT plays a more significant metabolic role than in humans, and are evolutionarily conserved (Urhammer et al. 1997). The fact may indicate a functional significance of the respective protein sites. Further studies showed that the Met229Leu substitution was more frequent in T2D patients than in healthy people from the Japanese population. In addition, Met229Leu was in linkage disequilibrium with the A–112C substitution (Mori et al. 2001). However, the association of the polymorphism with the disease was not confirmed in T2D patients from India. Yet the frequency of the haplotype [−3826A/−112C/229Met] was significantly higher in the T2D patients (Vimaleswaran et al. 2009).

### The effects of polymorphisms of the other *Ucp* family genes on metabolism

The *Ucp1* homologues, such as *Ucp2* and *Ucp3* also deserve attention among candidate genes whose polymorphisms may be associated with obesity and risk factors for cardiovascular diseases. *Ucp2* and *Ucp3* are in the q13 region of chromosome 11 and are 60% similar to *Ucp1.* The genes coding for UCP2 and UCP3 are involved in regulating free fatty acid metabolism and prevent the formation of reactive oxygen species, protecting cells from oxidative stress (Bondareva et al. 2018).

Obesity and T2D are often accompanied by the formation of reactive oxygen species in mitochondria and the accumulation of lipids in cardiomyocytes, skeletal muscle cells, and hepatocytes. Animal studies showed that lipids accumulated in cardiomyocytes exert cytotoxic effects and can lead to heart failure. An adaptive mechanism based on increased expression of *Ucp2* and *Ucp3* may be activated in some tissues, including the myocardium, to prevent oxidative stress and lipid toxicity. Being in excess, UCP2 and UCP3 neutralize the harmful effects of reactive oxygen species and reduce the accumulation of long-chain fatty acids, thus preventing cell death (Cadenas 2018; Ruiz-Ramírez et al. 2016).

UCP2 was shown to exert an anti-atherogenic effect on vessel walls (Blanc et al. 2003), thus improving tolerance to coronary heart disease (Cheurfa et al. 2008; McLeod et al. 2005), and to protect cardiomyocytes from death due to oxidative stress (Teshima et al. 2003). Most studies of the *Ucp2* and *Ucp3* polymorphisms focus on the SNPs that are in the *Ucp2* upstream region (G–866A (rs659366)), *Ucp2* exon 4 (Ala55Val (rs660339)), and the *Ucp3* promoter region (C–55 T (rs1800849)). These polymorphisms may be associated with T2D, obesity, fat metabolism, and mRNA expression (Wang et al. 2004).

### G–866A polymorphism of the *Ucp2* gene

**Fig. 3:**
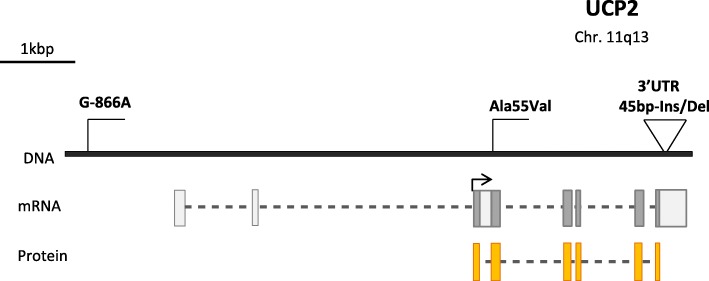
Schematic structure of the *Ucp2* gene and its best-studied polymorphisms

The G–866A polymorphism is upstream of the promoter of the *Ucp2* gene (Fig. 3) and affects the multifunctional cis-regulatory element that is responsible for the tissue-specific binding of various nuclear proteins (Esterbauer et al. 2001). The location of the polymorphism determines its influence on *Ucp2* expression. Studies showed that the minor A allele of the G–866A polymorphism increases *Ucp2* expression in vivo and enhances gene transcription in experiments with cell lines. A study of the G–866A substitution in obese and normal-weight patients showed that the A allele reduces the risk of obesity in middle-aged humans (Esterbauer et al. 2001). At the same time, such an association was not detected in children and adolescents (Le Fur et al. 2004; Oguzkan-Balci et al. 2013).

Increased *Ucp2* expression leads to a reduced ATP synthesis, which is among the factors that decrease insulin secretion and increase the risk of T2D. For example, healthy –866A allele carriers showed a decreased response to insulin when administered with glucose (D’Adamo et al. 2004; Krempler et al. 2002; Sasahara et al. 2004). A glucose tolerance test showed a decreased level of insulin secretion in –866A allele carriers with T2D (Gomathi et al. 2019), and insulin therapy was required for them more often and prescribed at earlier stages of the disease as compared with –866G allele carriers (D’Adamo et al. 2004). The –866A allele was associated with T2D in women aged 40 to 65 years (OR = 2.18, *P* < 0.05) in a study by Russian authors (Lapik et al. 2015). However, the opposite was demonstrated in several studies; e.g., the –866A allele was associated with reduced risk of T2D in the Italian population (Bulotta et al. 2005) and a decreased insulin sensitivity was detected in the GG genotype carriers (*P* = 0.05) in the Danish population (Andersen et al. 2013). The –866A allele was associated with a decrease in the conductivity of the peripheral nervous system in patients with T2D (Hiroshi et al. 2006) and was found to reduce the risk of diabetic neuropathy in type 1 diabetes (Rudofsky et al. 2006).

The G allele of the G–866A polymorphism is associated with reduced mRNA expression (Esterbauer et al. 2001; Sasahara et al. 2004), high BMI values, increased body fat mass (Yoon et al. 2007), high risk of obesity, and decreased risk of T2D (Krempler et al. 2002). Increased levels of triglycerides, total cholesterol, and LDL-C in the blood in –866G allele carriers were more frequent than in subjects carrying the –866A allele (Reis et al. 2004). On the other hand, the –866A allele was associated with obesity (*P* = 0.003) and hyperinsulinemia in the Indian population (Srivastava et al. 2010). The data are consistent with the results of a study by Wang et al., who showed that AA genotype carriers had decreased *Ucp2* mRNA levels relative to GG carriers (Wang et al. 2004). A study in Saudi Arabia showed that subjects with the AA and AG genotypes of the G–866A polymorphism were more prone to have nonalcoholic fatty liver disease (Mohseni et al. 2017).

Currently available data on the contribution of the G–866A polymorphism to the development of obesity and T2D are rather contradictory. However, the results of independent meta-analyses are generally consistent with each other. The –866A allele was shown to reduce the risk of obesity in European populations, but this association was not observed in Asians (Brondani et al. 2014b; Liu et al. 2013; Zhang et al. 2014a). Meta-analyses of studies of T2D did not reveal any association between the G–866A polymorphism and the disease among either Europeans and Asians (Qin et al. 2013; de Souza et al. 2013; Xu et al. 2011).

Because *Ucp2* is widely expressed in various tissue types, association of its polymorphisms are not limited to obesity and diabetes. As already mentioned, UCP2 reduces the production of reactive oxygen species, which are one of the main sources of oxidative stress and, as a consequence, may cause cell damage and death. Oxidative stress plays an important role in the pathogenesis of atherosclerosis and coronary heart disease. Studies associated the G–688A genetic substitution with cardiovascular pathologies. The –866A allelic variant was associated with reduced risk of coronary artery disease in French men with T2D (Cheurfa et al. 2008). AA genotype carriers who survived ischemic stroke showed a higher functional status as compared with GG and GA genotype carriers (Díaz-Maroto Cicuéndez et al. 2017). At the same time, the A allele increased the risk of developing coronary artery disease in the Mexican population (Gamboa et al. 2018) and in males of the European population (Dhamrait et al. 2004).

### Ala55Val polymorphism in exon 4 and Ins/Del 45 bp in the 3’UTR of the *Ucp2* gene

The Ala55Val polymorphism is in *Ucp2* exon 4, where a C/T nucleotide substitution leads to a substitution of alanine for valine at position 55 of the UCP2 amino acid sequence (Fig. 3). The results of studies of this polymorphism do not allow a clear conclusion about its association with obesity, T2D, and related metabolic disorders. A number of studies showed that Val/Val genotype carriers are at higher risk of T2D and obesity and have a higher BMI as compared with Ala/Val or Ala/Ala genotype carriers (Surniyantoro et al. 2018; Xiu et al. 2004; Yu et al. 2005). However, other studies found no association between the Ala55Val polymorphism and BMI, obesity, metabolic syndrome, T2D, and insulin secretion (Rosmond et al. 2002; Surniyantoro et al. 2018).

According to the results of meta-analyses (Brondani et al. 2014a; de Souza et al. 2011; Xu et al. 2011; Zhang et al. 2014a), the 55Val allele was more often associated with diseases in Asian populations.

The Ins/Del 45 bp polymorphism is in the 3’UTR of *Ucp2* exon 8 and is 158 bp downstream of the stop codon (Fig. 3). The insertion was shown to disturb the stability of the *Ucp2* mRNA in vitro (Esterbauer et al. 2001); i.e., the polymorphism is possibly of functional significance. However, this effect was not observed in vivo (Walder et al. 1998). The Ins allele frequency in obese patients was found to be significantly higher than in subjects with normal body weights. Ins allele carriers had higher BMI values, which increased the risk of obesity when the genome contained the Ins allele of *Ucp2* (Lee et al. 2008; Marti et al. 2004; Yanovski et al. 2000). The Ins/Ins genotype was associated with higher metabolic rates (*P* = 0.038), higher energy consumption, and lower BMI values in the Indian population (Walder et al. 1998). Del/Del genotype carriers were more efficient in reducing weight as compared with Ins/Del and Ins/Ins genotype carriers in the Japanese population (Mutombo et al. 2012). On the contrary, a weight loss program was more effective for Ins/Del and Ins/Ins genotype carriers in Caucasians of the Greek population (Papazoglou et al. 2012). Other studies found neither positive nor negative association between the Ins/Del polymorphism and changes in weight and BMI (Berentzen et al. 2005; Dalgaard et al. 1999; Maestrini et al. 2003; Papazoglou et al. 2012; Yiew et al. 2010).

The question of the biological significance of the Ins/Del polymorphism remains open today. It is of interest to study the effect of the insertion on the functionality of UCP2.

### *Ucp3* gene polymorphisms

**Fig. 4:**
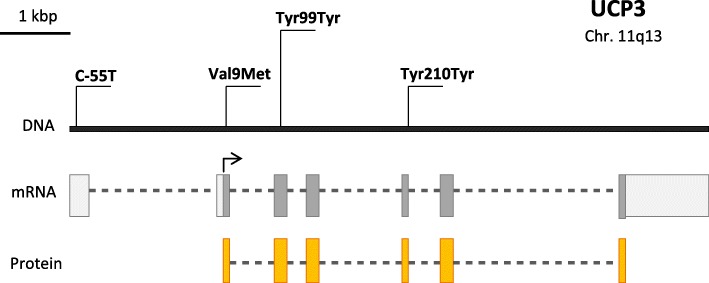
Schematic structure of the *Ucp3* gene and its best-studied polymorphisms

The most detailed data are available for the C/T nucleotide substitution at position − 55 (Fig. 4), which is in the *Ucp3* promoter region and is close to the presumed TATA box and 4 bp downstream of the presumed PPAR response element (Acín et al. 1999). The SNP location suggests an effect on transcription of the *Ucp3* gene.

Schrauwen et al. associated the − 55 T allele with increased expression of the *Ucp3* mRNA in skeletal muscles (Schrauwen et al. 1999b). *Ucp3* mRNA expression in muscle cells positively correlated with the resting metabolic rate (Schrauwen et al. 1999a). In Europeans and the Japanese, − 55 T allele carriers had lower BMIs (Halsall et al. 2001; Hamada et al. 2008; Lapice et al. 2014; Liu et al. 2005) and higher HDL-C levels (Hamada et al. 2008). The polymorphism inversely correlated with waist circumference in Mexican adolescents (Muñoz et al. 2017). However, opposite results were obtained in other studies. The BMI was higher in carriers of the − 55 T allele in Scandinavians (Lindholm et al. 2004). TT carriers showed higher BMIs in both obese patients and healthy controls in a study by French authors (Otabe et al. 2000). The results of a meta-analysis did not confirm the association between the polymorphism and the risk of obesity in Europeans or Asians (Qian et al. 2013). A study of the Chinese population similarly found no association between the polymorphism and obesity (Cai et al. 2017).

Contradictory results are similarly available for the association between the C–55 T substitution and the risk of T2D in different populations. The risk of T2D in − 55 T allele carriers was lower than in CC genotype carriers in the Brazilian (Schnor et al. 2017) and French (Meirhaeghe et al. 2000) populations, while no significant association with prediabetes was detected in the Chinese population (Su et al. 2018). Data from independent meta-analyses agree that the association of the C–55 T polymorphism with T2D is observed only in Asians (de Souza et al. 2013; Xu et al. 2011).

Less is known for the polymorphisms Val9Met (GTG/ATG) of exon 2, Tyr99Tyr (TAT/TAC) of exon 3, and Tyr210Tyr (TAC/TAT) of exon 5 of the *Ucp3* gene (Fig. 4). The TT genotype of the Tyr99Tyr polymorphism was weakly associated with diabetes, but not with obesity, in the French population (Otabe et al. 1999). A nearly statistically significant association of Tyr99Tyr with BMI and body fat mass was revealed by Lanouette et al. (Lanouette et al. 2002). The mutant alleles of the Tyr99Tyr and Tyr210Tyr polymorphisms were associated with high caloric intake and lean mass, and Tyr99Tyr was found to be in linkage disequilibrium with the C–55 T substitution (Damcott et al. 2004).

A population-based study of the *Ucp3 C*–55 T and *Ucp2* Ins/Del polymorphisms gave rise to a hypothesis about the role of the UCP family proteins in the regulation of angiotensin-converting enzyme (ACE). ACE is a component of the renin-angiotensin system, and acts to convert inactive angiotensin I into the active form angiotensin II, which causes constriction of blood vessels and thus increases the blood pressure. ACE is a target for a class of modern drugs that reduce the blood pressure by inhibiting the ACE molecule and thus blocking the formation of angiotensin II. A study conducted in patients with diabetes from Scandinavia and healthy UK residents revealed a link between the allelic variants of *Ucp3* –55C and *Ucp2* Ins and a higher level of ACE activity in the blood plasma. At the same time, a sixfold decrease in the activity of the *Ucp2* mRNA as a result of RNA interference was found to cause a parallel increase in *ACE* gene expression. On a more global scale, this hypothesis may lead to a revision of views on the ratio of extracellular and intracellular mechanisms of metabolic regulation. The applied medical value of such a mechanism may also be great. According to the authors of the article, these facts may provide direction for new research investigating the interface between local cellular metabolism and endocrine metabolic regulation (Dhamrait et al. 2016).

## Conclusions

A growing number of studies address the polymorphic variations in the *Ucp* gene family, and a major part of them focus on several polymorphisms of the *Ucp1* gene. The data sets concerning their association with CMD risk are largely mosaic and contradictory. An association of the polymorphisms with CMDs was not found in some European populations. However, most studies showed an associations with certain metabolic disorders. The discrepancy can be explained by small samples, ethnic differences, different climatic conditions in which the research was conducted, and differences in age and lifestyle. Because *Ucp* mutations are associated with disease and current research portrays conflicting findings, we need more thoughtfuly and carefully verify the studies in this field. In recent years, polymorphisms of the *Ucp2* and *Ucp3* genes have been increasingly included in the genetic factors to be analyzed. At the same time, the biological role of their genetic variations has not been fully understood or has been described based on only few in vitro studies. The lack of research on the biological role of genetic variations in the *Ucp* family largely influences the prognostic power in CMDs. In addition, data on the regulation of *Ucp* gene expression, signaling pathways and bioactive molecules involved in regulating the *Ucp* genes, and the role of polymorphisms in regulating the gene functions have just begun to accumulate. The greatest effect on gene expression is known for the polymorphisms of the regulatory gene regions, such as A–3826G in the 5′ noncoding region of *Ucp1*, G–866A in *Ucp2*, and C–55 T in the promoter of *Ucp3*. Many studies have associated these polymorphisms with the level of *Ucp1*–*Ucp3* gene expression, increased BMI, susceptibility to T2D, higher blood lipid levels, and elevated blood pressure. In some cases, the polymorphisms show an association with a disease only when considered together as components of a haplotype. Despite the great interest in the genes of the *Ucp* family, such studies have not been conducted at all in a number of Eastern European countries. Further research on the polymorphisms of the *Ucp* genes in representative samples of different ethnic groups and the accumulation of data on the biological role of genetic variations could have possible applications, such as treatment of metabolic syndrome.

## Data Availability

Not applicable.
